# Phytochemicals, promising strategies combating *Cutibacterium acnes*

**DOI:** 10.3389/fphar.2024.1476670

**Published:** 2024-12-09

**Authors:** Cuilian Sun, Yuhang Na, Ziyu Wang, Tingting Zhu, Xiaojuan Liu

**Affiliations:** ^1^ Department of Pathogen Biology, Medical College, Nantong University, Nantong, Jiangsu, China; ^2^ Medical College, Nantong University, Nantong, Jiangsu, China; ^3^ Department of Dermatology, The First Affiliated Hospital of Soochow University, Suzhou, China

**Keywords:** *Cutibacterium acnes*, acne vulgaris, phytochemicals, plant composition, antibiosis

## Abstract

The excessive proliferation of *Cutibacterium acnes (C. acnes)* is an important reason for the occurrence of acne vulgaris, and genetic detection ratio of *C. acnes* in acne is as high as 60.5%. Until now, the treatment of *C. acnes* is mainly limited to antibiotics, but some strains of *C. acnes* produce antimicrobial resistances, making it difficult for clinical treatment. Additionally, antibiotics can cause severe adverse effects. Therefore, more and more people are paying attention to phytochemicals. It is well known that plants can synthesize a range of secondary metabolites, named phytochemicals, part of which have antibacterial properties. Additionally, the main advantages of phytochemicals are that they have good efficacies and less side effects, so they are suitable choices for medical treatment. This review mainly discusses the effects and mechanisms of phytochemicals against *C. acnes*.

## 1 Introduction

Acne vulgaris (AV) is a chronic inflammatory disease of the pilosebaceous unit. The 2010 Global Burden of Disease Study found that AV ranked eighth in the common disease sequelae, with a global prevalence of 9.38% (Vos et al., 2012). Based on a systematic review and meta analysis about relevant studies published between 1 January 1996 and 30 September 2016, the overall pooled prevalence rates of acne in Mainland China were 39.2% (Li et al., 2017). AV is common in adolescents and can be identified by their facial lesion (Silverberg and Silverberg, 2014). It is mainly manifested as a series of clinical signs related to swelling, inflammation or scarring of sebaceous gland units (Ayer and Burrows, 2006), which can be divided into noninflammatory skin lesions (comedones) and inflammatory skin lesions. Noninflammatory lesions include open comedones (blackheads) and closed comedones (whiteheads). Inflammatory lesions can range from papules or pustules to nodules or cysts, and finally scar formation, which is the result of inflammation of the acne dermis, and these processes tend to occur on the face, neck, chest, and back of AV patients (Titus and Hodge, 2012). There are four primary factors in the pathogenesis of acne, which interact with each other to produce acne lesions, including inflammatory mediators released into the skin; alteration of the keratinisation process leading to comedones; sebum production by the sebaceous gland; and colonization by *C. acnes* (Thiboutot et al., 2009). The occurrence of acne may not cause serious harm physically, but it will cause psychological trauma to teenagers, causing low self-esteem, anxiety, social inhibition and depression (Williams et al., 2012).

*Cutibacterium acnes* is closely related to the occurrence of acne. The facial acne lesions of 375 acne patients were collected and 227 acne patients were isolated from *C. acnes*. The positive ratio of *C. acnes* was 60.5% (Zhu et al., 2019). *Cutibacterium acnes* is a lipophilic Gram-positive facultative anaerobic bacterium, possessing a typical appearance of corynebacterium, coarse and irregular short branched structure under microscope (Ahle et al., 2023). Excessive proliferation of *C. acnes*-induced Toll-like receptor (TLR)-2 and protease-activated receptors expressed in keratinocytes activate innate immunity, inducing the production of inflammatory factors and matrix metalloproteinases in early and advanced acne lesions (Williams et al., 2012). *Cutibacterium acnes* also stimulates increased sebum secretion, increased free fatty acids and abnormal keratinization of sebaceous ducts (Tucker et al., 1980). At the same time, the sebaceous glands of acne lesions provide an anaerobic and fat-rich environment for *C. acnes* to multiply within the sebum glands. In addition, *C. acnes* produces protease, lipase and hyaluronidase, which increases free fatty acids in acne lesions, stimulates excessive keratinization of sebaceous duct, resulting in poor sebum excretion, aggravates acne inflammation, and thus forms a vicious cycle to deteriorate acne (Adler et al., 2017). Moreover, besides acne, *C. acnes* causes severe infections at various body sites, resulting in endophthalmitis (Meisler and Mandelbaum, 1989), endocarditis (Banzon et al., 2017), central nervous system infections (Richards et al., 1989), arthroplastic and osteosynthetic infections (Lutz et al., 2005). Therefore, anti-*C. acnes* therapies are important in clinical practice tackling *C. acnes* associated diseases.

The common clinical approach to combat *C. acnes* is the use of topical or oral antibiotics, such as tetracyclines and macrolides. However, the long-term use of antibiotics can cause a dramatic increase in antimicrobial resistances (AMRs) (Habeshian and Cohen, 2020). Thus, it is not recommended the monotherapy of topical and oral antibiotics, which is replaced by antibiotics combined with benzoyl peroxide (BP). BP is an antibacterial agent that kills *C. acnes* through the release of free oxygen radicals, but BP therapy is limited by staining and bleaching of fabric, concentration-dependent irritation, dryness, erythema and uncommon contact allergy (Reynolds et al., 2024).

The phytochemicals have been used to ameliorate and cure diseases since ancient times, including the treatments of bacterial infectious diseases (Liang et al., 2022). Discovery of novel drugs can be accomplished with the use of the phytochemicals, which are the broad-spectrum secondary metabolites. The phytochemicals can effectively prevent and ease the toxicity induced by other toxins or drugs (Bhatia et al., 2021). Many studies have shown that some phytochemicals have anti-*C. acnes* efficacies (Sinha et al., 2014). These phytochemicals are classified as quinones, saponins, tannins, terpenoids, alkaloids, volatile oils, organic acids, phenols, and flavonoids according to the main active components. This review will summarize the ingredients efficiencies, and mechanisms of the phytochemicals that have anti-*C. acnes* effects.

## 2 The emerging threat of antibiotic therapy for *Cutibacterium acnes*

Currently, both topical and oral antibiotics are used to treat acne. Erythromycin and clindamycin, belonging to the antibiotic family of macrolides, are the two most commonly used topical antibiotics, and they can slow bacterial growth (Walsh et al., 2016). However, we have to face the problem of AMRs caused by the overuse and abuse of antimicrobial drugs. Many countries have reported that over 50% of *C. acnes* strains are AMRs (Walsh et al., 2016), and the incidence of *C. acnes* AMRs increased from 20% in 1978 to 62% in 1996 (Walsh et al., 2016). Approximately 50% of patients with acne develop resistance after oral or topical therapy, and 1 in 4 *C. acnes* strains are currently resistant to macrolides (Zhang et al., 2022). Although not specific to *C. acnes*, recent publications have highlighted the dangers of antibiotic resistance, the first comprehensive assessment on the global health impact of antimicrobial resistances (AMRs) in 2022 estimated that 4.95 million deaths in 2019 were associated with AMRs (Antimicrobial Resistance, 2022). The consequences of AMRs to *C. acnes* may include failure of acne treatment, skin microbiome disturbances, emergence of multidrug-resistant bacteria, and the spread of resistant strains to healthcare workers and the general population.

## 3 Possible alternatives to classical antibiotics

### 3.1 Bacteriophage therapy

Given the growing burden of AMRs worldwide, bacteriophage therapy is one of the promising solutions for AMRs (Strathdee et al., 2023). Phages are bacterial viruses widely distributed in environments. They attach to the surfaces of bacteria and inject their genomes into the bacterial cells, then manipulate the bacterial metabolic machinery to produce viral proteins and copy the viral genome. Finally, viral proteins and genes assemble to form new viral particles and the bacterial cells are lysed, releasing numerous new phages (Torres-Barceló, 2018). Many studies have shown that the use of phage can indeed reduce the number of *C. acnes* (Han et al., 2023; Rimon et al., 2023; Xuan G. et al., 2023), but bacteriophage therapy needs to face greater scrutiny in terms of safety and lack of efficacy data from clinical trials, and the low phage variability and phage resistance in *C. acnes* (Aslan Kayiran et al., 2020).

### 3.2 Antimicrobial peptides therapy

Antimicrobial peptides (AMPs) are positively charged amphiphilic molecules. Being electrostatically adsorbed on the surface of bacterial membranes, AMPs can easily penetrate and destroy the membrane structures of the bacteria, resulting in bacterial death (Xuan J. et al., 2023). Therefore, most AMPs exhibit a broad spectrum of antimicrobial activity. The effect of AMPs on *C. acnes* has been widely reported by far. Bombinin-like peptide 7 (BLP-7) has antibacterial activity with a minimum inhibitory concentration (MIC) of 5 μM, which was extracted from *Bombina orientalis* (Wu et al., 2020). Two novel AMPs WSKK11 and WSRR11 also provide great potentials to kill *C. acnes* (Theansungnoen et al., 2022). However, AMPs therapy still has shortcomings, including cause systemic and local toxicity, decreased sensitivity to salt, serum, and pH, sensitization and allergy after repeated application, confounding biological functions (e.g., angiogenesis) and high manufacturing costs (Gupta et al., 2018).

### 3.3 Phytochemicals therapy

The therapies bacteriophage and antimicrobial peptides bear high production costs and display low safety performance, easily causing local or systemic toxicity. Therefore, it is crucial to find a new therapeutic strategy that is easy to implement with little side effects. Phytochemicals have been studied as an alternative therapy to antibiotics. Traditional medicine has used plants to fight diseases for thousands of years, and according to World Health Organization (WHO), 80 percent of the global populations rely on the plant-based medicines for their basic healthcare needs (Kifle et al., 2021). In fact, many reports have pointed to the possibility of antimicrobial use of medicinal plant active ingredients. For example, Ginger essential oil (GEO) inhibits and kills *Escherichia coli* (*E.coli*) and *Staphylococcus aureus* (*S. aureus*) (Wang et al., 2020). These important bioactive compounds have broad application prospects in the field of biomedical development.

Now the inhibitory effects of phytochemicals, such as phloretin (Cheon et al., 2019), and rhodomyrtone (Saising and Voravuthikunchai, 2012), on *C. acnes* have been widely studied. Phytochemicals are readily available, effective, and with small numbers of side effects, and are expected to be widely used. Therefore, it is necessary to study the inhibitory effects and underlying mechanisms of phytochemicals on *C. acnes*.

## 4 Phytochemicals that have an inhibitory effect on *Cutibacterium acnes*

Considerable efforts have been made to find plant-derived antimicrobials against C. acnes. Table 1 summarizes 31 phytochemicals with anti-C. acnes activity, showing their classification, botanical source, MIC, minimum bactericidal concentration (MBC), and tested C. acnes strains. These phytochemicals with anti-C. acnes activities were either isolated from plants or commercially purchased as standard reagents. In view of the antibacterial activity parameters (Kuete, 2010), including significant (MIC ≤10 μg/mL), moderate (MIC ≤100 μg/mL) and low or negligible (MIC ≤100 μg/mL), 17 phytochemicals have significant inhibitory effects, and the hop extract Lupulones has the strongest anti-C. acnes ability.

**TABLE 1:** Summary of phytochemicals with anti-*C. acnes* s activity.

Phytochemicals	Classification	Botanical source	Botanical Family	Plant part used	Bacterial Strain	MIC/MBC (μg/mL)	References
Resveratrol	Polyphenols	Smilax china L.	Liliaceae	Root	KCTC 3314	125/125	Joo et al. (2022)	
Quercetin	Flavonoid	Smilax china L.	Liliaceae	Root	KCTC 3314	31.25/31.25	Joo et al. (2022)	
Kaempferol	Flavonoid	Impatiens balsamina	Balsaminace-ae	Whole plant	ATCC 6919	≤32/≤128	Lim et al. (2007)	
Thymol	Phenols	Origanum vulgare L.	Labiatae	Whole plant	ATCC 6919	700/1,400	Taleb et al. (2018)	
Honokiol	Biphenols	Magnolia sp.	Magnoliaceae	Stem bark	ATCC 6919	3–4/20	Park et al. (2004)	
Magnolol	Biphenols	Magnolia sp.	Magnoliaceae	Stem bark	ATCC 6919	9/45	Park et al. (2004)	
Rhodomyrtone	Phenols	Rhodomyrtus tomentosa (Aiton) Hassk	Myrtaceae Juss.	Leaves	Acne lesions from human volunteers	0.12–0.5/0.12–1	Saising et al. (2012)	
Acidic polysaccharide CS-F2	Polysaccharie	Camellia sinensis	Theaceae	Whole plant	ATCC 6919	50/-	Lee et al. (2006)	
Sapindoside A	Triterpenes	S. mukorossi	Arboricaceae	Whole plant	ATCC 6919	6.3/-	Wei et al. (2022)	
Sapindoside B	Triterpenes	S. mukorossi	Arboricaceae	Whole plant	ATCC 6919	13/-	Wei et al. (2022)	
Punicalagin	Tannins	Punica granatum Linne	Punicaceae	Pericarp	BCRC10723	6.25/12.5	Lee et al. (2017)	
Punicalin	Tannins	Punica granatum Linne	Punicaceae	Pericarp	BCRC10723	6.25/12.5	Lee et al. (2017)	
Strictinin A	Tannins	Punica granatum Linne	Punicaceae	Pericarp	BCRC10723	12.5/25	Lee et al. (2017)	
Granatin B	Tannins	Punica granatum Linne	Punicaceae	Pericarp	BCRC10723	100/-	Lee et al. (2017)	
Lauric acids	Saturated fatty acid	Coconut	Palmae	Oil	ATCC 6919	−/−	Darren Yang et al. (2009)	
Myrtucommulone A	Phloroglucino-ls	Myrtus communis L.	Myrtaceae	Leaves	EryR (EryS)	1.2 (1.2)/-	Feuillolay et al. (2016), Fiorini-Puybaret et al. (2011)	
Myrtucommulone B	Phloroglucino-ls	Myrtus communis L.	Myrtaceae	Leaves	EryR (EryS)	0.3 (0.6)/-	Fiorini-Puybaret et al. (2011)	
Humulones	Hop alpha acid	Humulus lupulus Linn.	Moraceae	Flowers	ATCC 6919	10/30	Yamaguchi et al. (2009)	
Lupulones	Hop beta acid	Humulus lupulus Linn.	Moraceae	Flowers	ATCC 6919	0.1/0.3	Yamaguchi et al. (2009)	
Xanthohumol	Flavonoid	Humulus lupulus Linn.	Moraceae	Flowers	ATCC 6919	3/3	Yamaguchi et al. (2009)	
Icariin	Flavonol	Epimedium brevicornum	Berberidace-ae	Whole plant	LMG 16711	25/-	Coenye et al. (2012)	
Alpha-kosin	Long chain fatty alcohols	Leucosidea sericea	Rosaceae	Leaves	—	1.9/-	Sharma et al. (2014)	
Artocarpin	Flavonoid	Artocarpus integer	Moraceae	Root	DMST 14916	2/32	Dej-adisai et al. (2013)	
Cudraflavone C	Flavonol	Artocarpus integer	Moraceae	Root	DMST 14916	2/32	Dej-adisai et al. (2013)	
Artocarpanone	Flavonoid	Artocarpus integer	Moraceae	Root	DMST 14916	32/64	Dej-adisai et al. (2013)	
5,7-dimethoxyflavo-ne	Flavonoid	Kaempferia parviflora Wall. ex Baker	Zingiberace-ae	Rhizome	DIMST 14916	−/−	Sitthichai et al. (2022)	
Pulsaquinone	Quinone-type compound	Pulsatilla koreana	Ranunculac-eae	Rhizomes	KCTC 3314	2/-	Cho et al. (2009)	
Farnesol	Sesquiterpene alcohol	Magnolia flower, loquat leaves and other plants	—	Flowers, leaves, stems, etc	—	0.14 mM^a^/-	Wu et al. (2021)	
Dumoside	C22-steroidal lactone glycosides	Paris polyphylla var. yunnanensis	Trilili aceae	Stems and leaves	—	3.9/-	Qin et al. (2013)	
15-nor-14-oxolabda-8 (17),12E-diene-18-oic acid	Diterpene aldehyde	Pinus densiflora	Pinaceae	Cones	—	64/-	Sultan et al. (2008)	
Phloretin	Flavonoid	Apples and other plants	—	Branches, leaves, etc	ATCC 11827	500/-	Kum et al. (2016)	

^a^ The MIC of Farnesol was 0.14 mM, and the concentration unit was not µg/mL (Wu et al., 2021).

In the following section, the inhibitory effects of phytochemicals on C. acnes and their mechanisms will be described in detail according to the classification of plant active ingredients, including phenols, flavonoids, Polyols, essential oils, tannins, saponins, fatty acids and Quinone compounds.

### 4.1 Phenols and their derivatives, polyols and flavonoids

Phenols, Polyols and flavonoids are widely distributed in the plant kingdom, which have antioxidant, anti-inflammatory, antibacterial and antiviral activities. Antibacterial mechanisms of flavonoids including inhibition of nucleic acid synthesis, cytoplasmic membrane function and energy metabolism (Cushnie and Lamb, 2005).

Resveratrol (RSL) is a polyphenolic compound derived from the Smilax plant, peanuts, blueberries and cranberries, and it is an antioxidant with a variety of biological effects, including antibacterial properties. Studies have shown the beneficial effects of RSL in fighting C. acnes (Docherty et al., 2007; Taylor et al., 2014). After RSL treatment, C. acnes shows changes in bacterial morphology under electron microscope, represented by intracellular edema and destruction of intracellular structural integrity (Taylor et al., 2014). As a membrane permeable compound, RSL may be able to alter the bacterial membrane structure of C. acnes and disrupt intracellular machinery (Taylor et al., 2014). At present, the specific mechanism of resveratrol inhibiting *C. acnes* is not clear, but studies have shown that RSL can bind to *E. coli* adenosine triphosphate (ATP) synthase and inhibit ATP hydrolysis and synthesis (Dadi et al., 2009). Filamentous temperature sensitive protein (FtsZ, a GTP-dependent prokaryotic cell division protein) is a key protein in septum formation during cell division, and it is responsible for cell division by forming a dynamic Z ring in the middle of the cell. In *E. coli*, RSL can inhibit FtsZ-mediated Z ring formation and inhibit cell division (Hwang and Lim, 2015; Ma et al., 2018). Furthermore, RSL can also alter the expression of bacterial virulence characteristics, thereby reducing toxin production, inhibiting biofilm formation, reducing motility, and interfering with quorum sensing (Vestergaard andIngmer, 2019). These might be the mechanisms for anti-*C. acnes* activities of RSL.

Honokiol (HON) and magnolol (MAG) are two major phenolic constituents of Magnoliaceae, which have anti-*C. acnes* activities (Park et al., 2004). In addition, they have anti-inflammatory effects and can be used as acne-mitigating candidates for topical application (Park et al., 2004). HON and MAG have effective antibacterial effects against Gram-positive bacteria, showing inhibitory effects on the bacterial thioredoxin (Trx) system (the main thiol-dependent disulfide reductase system in bacteria) (Ouyang et al., 2023). Most Gram-positive bacteria have a Trx system but lack a glutathione (GSH)/glutaredoxin (Grx) system, so in these Gram-positive bacteria, the Trx system plays irreplaceable roles in DNA synthesis and repair, cell proliferation and antioxidant defense. The inhibition of Trx by HON and MAG can disrupt the intracellular redox environment of bacteria. HON and MAG lead to dysregulation of cellular redox environment homeostasis and increase intracellular ROS levels, eventually contribute to their bactericidal efficacies (Ouyang et al., 2023).

Phloretin (PHL) is a flavonoid found in free form and glycoside form in apples and strawberries, with antioxidant, anti-inflammatory and antibacterial activities. H. Kum et al. demonstrate that PHL has inhibitory effects on *C. acnes*, *propionibacterium granulosum* and *Staphylococcus epidermidis (S. epidermidis) (* Kum et al., 2016). Fatty acids are essential for bacterial survival, hence molecules involved in fatty acid synthesis can be targeted by antimicrobial agents (Cheon et al., 2019). β-ketoacyl acyl carrier protein synthase III (KAS III) is one of the important enzymes in bacterial fatty acid synthesis. PHL can bind to the active site of *C. acnes* KAS III, block the extension reaction between *C. acnes* KAS III and its substrate, and finally inhibit the synthesis of fatty acids in *C. acnes* to obtain antibacterial effect (Cheon et al., 2019; Lee et al., 2009).

Erythritol is a kind of sugar alcohol compound, belonging to the polyalcohol sweetener, which is widely found in fruits such as melons, grapes and various fermented foods. Erythritol acts as an antioxidant *in vivo* and may prevent blood vessel damage caused by hyperglycemia (den Hartog et al., 2010). It also has an antibacterial effect, including *Streptococcus gordonii* (Hashino et al., 2013), *Corynebacterium minutissimum*, *Corynebacterium striatum*, and *S. epidermidis* (Fujii et al., 2022a). Fujii T et al. demonstrated that erythritol contributed to the growth of *C. acne*s ribotype 2 (RT2) and RT6 associated with healthy skin, inhibiting RT4 and RT5 associated with the progression of acne (Tadashi Fujii et al., 2022b). Glycerol 3-phosphate dehydrogenase encoded by g3pD is one of the key enzymes in erythritol metabolism. Due to the low expression level of g3pD in RT5, phosphorylated erythritol might accumulate intracellularly in the presence of erythritol in the environment, thereby directly inhibiting the activity of glycolytic enzymes (Tadashi Fujii et al., 2022a). Xylitol is a sugar alcohol compound with one more carbon atom than erythritol. L Trahan reveals that the antibacterial effect of xylitol is mainly through direct inhibition of the phosphoenolpyruvate phosphotransferase system (PEP-PTS), which causes the accumulation of xylitol-derived xylitol 5-phosphate inside bacterial cells, thus inhibiting the activity of glycolytic enzyme (Tadashi Fujii et al., 2022b; Trahan, 1995). Erythritol may have a growth inhibiting effect on C. acnes similar to xylitol (Tadashi Fujii et al., 2022a).

### 4.2 Volatile oil

Volatile oil, also known as essential oil, is a general term for an oily liquid that can be volatilized at room temperature and can be distilled with steam. The basic compositions of volatile oil include aliphatic compounds, aromatic compounds and terpenoids. In addition, it also contains other compounds, such as ligustilide, a phenol component in Angelica. Volatile oil is an important active ingredient, which can be directly used in the clinical application of crude drugs mainly containing volatile oil (Vora et al., 2024).

Oregano essential oil (OEO) contains thymol, carvacrol and ursolic acid, which is a kind of Volatile oil. OEO plays a spectral antibacterial activity, especially on *C. acnes* (Taleb et al., 2018). After OEO treatment, the bacteria mainly show protrusions on the cell surface without typical bacterial morphology, and the number of bacteria decreases (Scandorieiro et al., 2022). Though the specific antibacterial mechanisms of OEO against *C. acnes* are occult, OEO can inhibit the production and activity of lipase and coagulase of *S. aureus*, thus down-regulating the growth of bacteria (Soltani et al., 2021). In addition, OEO, as an anti-biofilm agent, has broad-spectrum anti-biofilm activity, which can inhibit and destroy the formation of *Streptococcus pyogenes* and *Enterococcus faecalis* biofilms (biofilm is composed of bacterial clusters and extracellular polymer matrix, ensuring the survival environment of bacteria) (Ogawa et al., 2011; Sirati et al., 2024; Wijesundara and Rupasinghe, 2018; Zhan et al., 2022). Ultee A et al. reveal that in *Bacillus cereus*, thymol and carvacrol, as ingredients of OEO, can damage cellular membranes and reduce the pH gradient in the cellular membranes, resulting in proton motive force, reduced ATP pools, and cell death (Ultee et al., 2002). Therefore, it is speculated that OEO may play its anti-C. acnes role via the above mechanisms.

### 4.3 Tannins

Tannins are a kind of polyphenolic compounds with complex structure in plants, which can be divided into hydrolyzable tannins and condensed tannins according to their chemical structures. Many tannins have pharmacological activities. The tannins contained in Cyrtomium fortunei have strong inhibitory effect on a variety of influenza viruses, the tannins contained in Castanea sativa mill leaf are effective substances in the treatment of acne (Piazza et al., 2024), and some tannins also can be used as effective antibacterial agents for *C. acnes*.

Punicalagin (PUN) is a tannin compound isolated from Punica granatum Linne (Lee et al., 2017). It is a low polymeric compound of 2-3 ellagic acids and can be degraded to produce ellagic acid. PUN has anti-inflammatory, antioxidant and antibacterial properties (Lee et al., 2017). Studies have revealed that PUN can destroy the cell structures of *C. acnes*. The bacterial surface exhibits obvious shrinkage and the surrounding environment is damaged under the electron microscope after 12 h treatment with PUN (Lee et al., 2017). In addition, PUN also has the effect of inhibiting lipase, which is an enzyme that hydrolyzes lipids. Many bacteria, such as *C. acnes*, can secrete lipase to decompose lipids and promote the absorption of lipid-related nutrients from external media, thus benefiting the growth of bacteria itself. The fluorescence determination of the lipase treated by PUN shows that PUN can inhibit the lipase activity of *C. acnes* (Lee et al., 2017). Moreover, Bakkiyaraj D et al. show that ellagic acid, a degradation product of PUN, has anti-biofilm potential against a large number of pathogenic bacteria (Bakkiyaraj et al., 2013).

### 4.4 Saponins

Saponins are a special class of glycosides existing in the plant kingdom. According to the structure produced by hydrolysis, saponins are divided into two categories, including triterpenoid saponins and steroid saponins. Saponins have anti-inflammatory, antibacterial and anti-tumor effects, having pharmacological activities in clinical practice (Passos et al., 2022).

Sapindus saponins are a group of Saponins, which extracted from Sapindus mukorossi Gaertn (S. mukorossi). S. mukorossi is a wide distributed medicinal plant. The Compendium of Materia Medica reports that S. mukorossi extract traditionally can be used to treat cough, excess salivation and whitening skin (M. P. Wei et al., 2021). Sapindus saponins have antibacterial (Alberice et al., 2012), antifungal (Hu et al., 2018), antitumor (Kuo et al., 2005) biological activities. Wei MP et al. point out that Sapindus saponins have anti-*C. acnes* activity (Wei et al., 2021). They mainly investigate the synergistic antibacterial mechanism of two saponins [saponins A and B (SAB)] against *C. acnes* 6919. The compositions of fatty acids in the cell membrane of *C. acnes* are changed with the treatment of SAB. 12-methyl-tetraalkanoic acid and octadecanoic acid decreases and increases, respectively. Meanwhile, the genes related to fatty acid biosynthesis are significantly downregulated. The changes of fatty acid composition in the membrane of *C. acnes* increase the hydrophobicity of the cell surface and decrease the fluidity of the membrane (Wei et al., 2021). In addition, molecular docking calculations show that SAB interacts with Malonyl-CoA-acyl carrier protein transacylase (FabD; an essential enzyme for bacterial type II fatty acid synthesis), through hydrogen bonding and hydrophobic interactions, thus SAB have a competitive inhibitory effect on fatty acid biosynthesis (Wei et al., 2021). In summary, SAB alter the fatty acid composition of *C. acnes* and further disrupt cell membrane properties, suggesting that Sapindus saponins may be natural additives against *C. acnes*.

P. polyphylla saponins, the mixture of saponins extracted from Paris polyphylla, have spectral antibacterial activity and have been shown to inhibit *C. acnes*, *S. aureus* and *S. epidermidis* (Thapa et al., 2022). The MIC of P. polyphylla saponins against *C. acnes* NCTC737L and *C. acnes* ATCC6919 are 97.5 μg/mL and 48.7 μg/mL, MBC are 198 μg/mL and 97.5 μg/mL, respectively (Li et al., 2024). Among the known saponins extracted from P. polyphylla, including PP I, PP II, PP VI, PP H and PP VII, PP I has the strongest antibacterial effect, while PP H has a relatively weak antibacterial effect (Li et al., 2024). However, the mechanism of P. polyphylla saponins against *C. acnes* need to be further explored.

### 4.5 Fatty acid

Fatty acids are divided into essential fatty acids and non-essential fatty acids according to whether they can be synthesized by the body. Non-essential fatty acids are fatty acids that can be synthesized by the body without relying on the food supply, and they include saturated fatty acids and some monounsaturated fatty acids. Essential fatty acids are essential for human health and life, but the body can’t synthesize it itself and must rely on the food supply, they are unsaturated fatty acids. Fatty acids are closely related to growth and development, intellectual development, memory and physiological functions (Yashodhara et al., 2009). Such as linoleic acid and arachidonic acid can promote the transport of cholesterol in the blood and reduce its deposition on the blood vessel wall (Demetz et al., 2014). Besides, some fatty acids can inhibit the growth of bacteria (Lamas et al., 2019).

Lauric acid (LA), a natural free fatty acid, which is the main acidic compound in coconut oil, has anti-*C. acnes* activity and can completely kill *C. acnes* at 80 μg/mL (Yang et al., 2009). LA may kill bacteria by breaking down the cell membrane of Gram-positive bacteria while the cell wall of the bacteria is intact, leading to the disintegration of the bacterial cytoplasm (Bergsson et al., 2001). LA is difficult to be dissolved in water. Therefore, to dissolve LA into creams and gels for better application, Darren Yang loads LA into liposomes and prepares LipoLA (Yang et al., 2009). LipoLA also has anti-*C. acnes* activity similar to that of LA. LipoLA was labeled by FRET chromophobe, and the change of FRET signal when LipoLA mixed with bacteria under different conditions was monitored to detect the bacteriostatic mechanism. The data suggest that LipoLA fuses with the membrane of *C. acnes* and releases the carried LA into the bacterial membrane, which is consistent with the bacteriostatic mechanism of LA (Yang et al., 2009).

### 4.6 Quinone compound

Quinones are mainly divided into four types, benzoquinone, naphthoquinone, phenanthrene quinone and anthraquinone, which usually have anti-cancer, anti-bacterial and anti-reactive oxygen activities.

Shikonin (SHI) is a naphthoquinone compound extracted from the root of Lithospermum erythrorhizon Siebold and Zucc, which has been widely used in Asia, and is currently believed to have less obvious side effects, and has anti-tumor (Wang et al., 2024), anti-viral (Borde et al., 2023), antioxidant (Gautam et al., 2024) and antibacterial (Watson et al., 2023) effects. A recent study explores the effects of SHI on *C. acnes* strains ATCC 6919 and KCCM 42791. SHI inhibits the growth and biofilm formation of *C. acnes* in a dose-dependent manner (Kim et al., 2024). The study also shows that SHI reduces biofilm formation by decreasing the production of extracellular polymeric substances (EPS) and increasing the production of porphyrin in *C. acnes* (Kim et al., 2024). In addition, quantitative real-time PCR (qRT-PCR) displays that the expression of 11 genes related to biofilm and virulence in *C. acnes* is changed after the application of SHI. For example, the mRNA levels of adhesin, lipase, hyaluronate lyase and virulence related genes is downregulated (Kim et al., 2024). ATP production is essential for bacterial survival and related functions, and inhibition of ATP synthase destroys cellular energy and leads to bacterial death. Zulfiqar Ahmad et al. propose that SHI inhibit the ATP synthase of *E. coli*, thus inhibiting the activity of *E. coli*, which may also be one of the reasons why SHI play antibacterial roles, including against *C. acnes* (Watson et al., 2023).

## 5 Combination therapy

Many phytochemicals have shown good anti-*C. acnes* ability, but some studies have shown that the combination of some phytochemicals with other substances will have better bactericidal properties. Lim et al. studied the efficacy of quercetin and kaempferol combined with two commonly used antibiotics (erythromycin and clindamycin) against *C. acnes* and found that the bactericidal activity of quercetin or kaempferol was significantly increased when combined with clindamycin (Lim et al., 2007). Taylor et al. evaluated the effect of resveratrol in combination with BP on C. acnes. Resveratrol showed sustained antibacterial activity against C. acnes, while BP showed a short-term bactericidal response. The combination of resveratrol and BP showed high initial antibacterial activity and sustained bacterial growth inhibition, with better antibacterial activity (Taylor et al., 2014).

## 6 Conclusion and prospect

**FIGURE 1:**
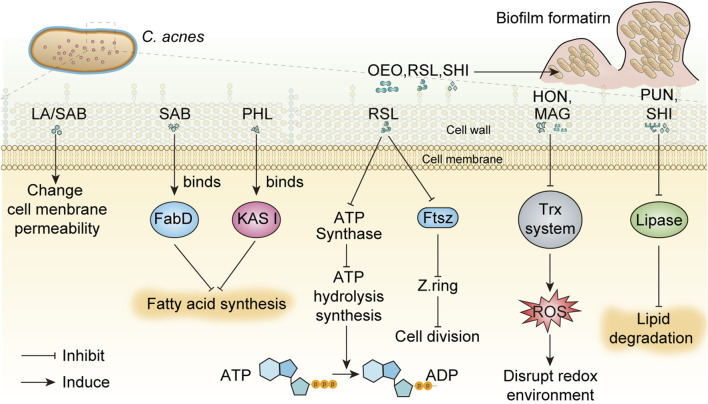
The mechanisms of phytochemicals against *C. acnes* were summarized. In this figure, the inhibition of ATP synthase and Ftsz protein by RSL on *C. acnes* was speculated by the inhibition mechanism of RSL on *E. coli*, which has not been confirmed by relevant studies. Oregano essential oil has broad-spectrum anti-biofilm activity, but its anti-*C. acnes* biofilm effect is only speculated and has not been confirmed. Abbreviations in the Figure: ADP, adenosine diphosphate; ATP, adenosine triphosphate; FabD, Malonyl-CoA-acyl carrier protein transacylase; FtsZ, filamentous temperature sensitive protein; HON, honokiol; KAS III, β-ketoacyl acyl carrier protein synthase III; MAG, magnolol; resveratrol, RSL; SAB, saponins A and B; SHI, shikonin; Trx, thioredoxin; LA, Lauric acid; OEO, oregano essential oil; PHL, Phloretin; PUN, punicalagin.

Overgrowth of *C. acnes* produces extracellular enzymes and specific inflammatory factors that break down triglycerides in sebum into free fatty acids and glycerol, leading to worsening of acne. A large number of studies have suggested that phytochemicals, including phenolic compounds, flavonoids, Polyols, essential oils, tannins, saponins, fatty acids and Quinone compounds, have inhibitory effects on *C. acnes*. The mechanisms of these components against *C. acnes* mainly include changing membrane permeability, inhibiting protein and nucleic acid synthesis, inhibiting enzyme activity, controlling pathogenic bacteria and preventing biofilm formation (Figure 1). However, current studies lack detailed investigation or demonstration of these mechanisms, which may reduce the depth of scientific analysis. Among the phytochemicals discussed in this review, myrtucommulone B and lupulones show extraordinary activity with MIC of 0.3 μg/mL and 0.1 μg/mL, followed by rhodomyrtone with MIC of 0.12–0.5 μg/mL.

In addition to the phytochemicals mentioned above, there may be numerous phytochemicals in nature that have better inhibitory activity against *C. acnes*, which have not been discovered until now. For example, cannabinoids have inhibitory activities against a variety of Gram-positive bacteria, including *Listeria monocytogenes*, *S. aureus* and so on (Saleemi et al., 2022). Cannabinoids can act on the membrane of bacterial cells, change the membrane permeability, and block the release of bacterial vesicles (Saleemi et al., 2022). We hypothesize whether cannabinoids may also have inhibitory effects on C. acnes, which needs further exploration.

Some phytochemicals have anti-*C. acnes* activity due to their phytoactive ingredients. These ingredients can be used as alternative candidates for drug development to alleviate AMRs and adverse drug reactions.

However, most current research relies on vitro experiments to demonstrate the efficacy of phytochemicals against *C. acnes*. *In vivo*, the studies mainly prove the anti-inflammatory and anti-lipid effects of phytochemicals, lacking phytochemicals anti-*C. acnes* experiment validation. In other words, their antibacterial effects haven’t been authenticated *in vivo*, and their therapeutic potential hasn’t been validated using clinical trials yet. Therefore, further investigation is needed in this field. In addition, in the practical application of anti-*C. acnes*, the stability and bioavailability of phytochemical in the acne treatment formula are key factors to achieve the therapeutic effect. pH value, temperature, light, metal ions, and co-pigments can directly affect the stability of phytochemicals (Kumar et al., 2023). Through studying the stability and bioavailability of these ingredients, more effective and safer acne treatment formulations can be developed. For example, the scientists package and deliver phytochemicals using nanoparticle drug delivery systems, leading to enhanced stability and bioavailability of phytochemicals (Melim et al., 2022).
